# Assessment of industrial pollution and water quality in the Nile River using GIS-based indices at Aswan, Egypt

**DOI:** 10.1038/s41598-025-33738-5

**Published:** 2025-12-31

**Authors:** Ahmed N. A. Abdou, Mohamed Hamed, Abdelmonsef M. M. Hassan, Mostafa A. Khaled

**Affiliations:** 1 Aquatic Ecology Department, Faculty of Fish and Fisheries Technology, Aswan University, Aswan, Egypt; 2 Department of Zoology, Faculty of Science, Al-Azhar University (Assiut Branch), Assiut, 71524 Egypt; 3 Department of Comparative Biomedical Sciences, School of Veterinary Medicine, Louisiana State University, Skip Bertman Drive, Baton Rouge, LA 70803 USA; 4 Egyptian Environmental Affairs Agency (EEAA), Aswan, Egypt; 5 Marine Science Department, National Authority for Remote Sensing &Space Sciences (NARSS), Cairo, Egypt

**Keywords:** GIS, Industrial effluent, Nile River, Spatial distribution, Water quality indices, Ecology, Ecology, Environmental sciences, Hydrology, Water resources

## Abstract

Industrial pollution represents a critical threat to freshwater ecosystems, particularly in regions where rivers serve multiple socio-economic functions. The Nile River in Aswan Governorate, southern Egypt, is a vital source of drinking water, irrigation, fisheries, and tourism, yet it is increasingly exposed to untreated industrial effluents. This study assessed the spatial extent and intensity of such pollution using geographic information system (GIS)-based spatial interpolation alongside two comprehensive indices: the weighted arithmetic water quality index (WAWQI) and the canadian water quality index (CWQI). Findings revealed distinct pollution hotspots near major industrial discharge areas, where water quality was classified as poor to very poor (WAWQI: 52.6–127.4; CWQI: 74.2–68.3). In contrast, upstream and downstream zones more distant from effluent sources exhibited good water quality (WAWQI: 24.5–38.1; CWQI: 93.69–85.52). The most degraded sites were characterized by elevated concentrations of chemical oxygen demand (13.1–59.6 mg/L), biochemical oxygen demand (7.67–36.5 mg/L), total suspended solids (4.67–13.1 mg/L), turbidity (6.01–17.1 NTU), total nitrogen (1.13–3.35 mg/L), and phenol (8.01–10.10 µg/L). These results highlight the ecological vulnerability of the Nile River in Aswan to industrial activities, with direct implications for ecosystem health and resource sustainability. They also underscore the limitations of relying solely on traditional water quality monitoring without spatial analysis, as GIS-based interpolation proved critical in detecting pollution gradients beyond sampling sites. The study supports the enforcement of Egypt’s Law 48/1982 on water protection and contributes to global sustainable development goals, particularly SDG 6 (Clean Water and Sanitation), SDG 12 (Responsible Consumption and Production), and SDG 14 (Life Below Water).

**Supplementary Information:**

The online version contains supplementary material available at 10.1038/s41598-025-33738-5.

## Introduction

Industrial pollution is one of the leading drivers of freshwater degradation worldwide, with rivers in both developed and developing regions experiencing significant ecological stress. Effluents from industries such as textiles, paper, sugar, tanneries, and metallurgy often contain high loads of organic matter, nutrients, heavy metals, oils, and synthetic chemicals that exceed the assimilative capacity of aquatic ecosystems ^1,2^. In Asia, major rivers like the Ganges, Yamuna, and Yangtze are heavily impacted by industrial discharges, resulting in oxygen depletion, toxic contamination, and the decline of fish populations ^3^. Similarly, in Africa and Latin America, rapid industrialization coupled with weak wastewater treatment infrastructure has exacerbated pollution in rivers such as the Niger, Congo, Nigeria and Amazon, with downstream communities facing heightened risks to health and livelihoods ^4,5^. Even in Europe and North America, where regulatory frameworks are stronger, legacy pollution from mining, metallurgy, and petrochemicals continues to impair water quality in rivers like the Danube and Mississippi ^6^. Globally, the cumulative effect of industrial effluents threatens biodiversity, disrupts food webs, undermines water security, and poses long-term risks to human health, underscoring the urgent need for stricter enforcement of environmental laws, adoption of cleaner technologies, and investment in sustainable wastewater management ^6^.

The Nile River is crucial for the United Nations Sustainable Development Goals, including clean water and sanitation (SDG 6). Effective management of the Nile River is essential for ensuring water security, ecosystem health, and long-term socio-economic stability in Egypt. However, this goal faces mounting challenges due to population growth, rapid urbanization, hydropower development, untreated wastewater discharge, and climate change ^7,8^. The discharge of untreated or poorly treated effluents into freshwater bodies poses a serious environmental threat, underscoring the need to adopt sustainable wastewater treatment and pollution control strategies ^9^. Water quality is a fundamental environmental factor that influences ecosystem functioning, public health, and sustainable development outcomes ^10–12^. In the Aswan Governorate of Upper Egypt, the Nile River receives substantial volumes of untreated industrial wastewater, particularly from sugar factories, paper mills, and a ferroalloy plant ^13^. Sugar and paper industries are highly water-intensive and discharge significant amounts of effluent into the environment, often containing high levels of organic matter, suspended solids, nutrients, and oils ^14–16^.

Alongside industrial discharge, the river is increasingly impacted by agriculture, fishing, oil spills, and untreated sewage ^17^. In southern Egypt, wastewater from industrial and domestic sources has already had long-lasting negative effects on water quality ^18^. Previous studies using water quality indices suggest that while the Nile retains some self-purification capacity, water in many sections is classified as poor for drinking, marginal for aquatic life, and only fair for irrigation ^19^. By contrast, Lake Nasser demonstrates relatively favorable physicochemical properties^20^.

Pollution in the Nile is the result of multiple, interacting stressors. Agricultural runoff contributes fertilizers and pesticides that accelerate eutrophication ^21,22^, while oil spills and solid waste further reduce water quality ^23^. Downstream sections of the river are particularly vulnerable due to the cumulative build-up of pollutants and reduced dilution capacity ^24^. Addressing these issues requires integrated watershed management, strict enforcement of environmental regulations, and sustainable land- and water-use practices to protect and restore ecological health.

Modern technologies such as geoinformatics provide powerful tools for addressing these challenges. By integrating geographic information systems (GIS), remote sensing (RS), and spatial analysis, geoinformatics allows for the monitoring, mapping, and evaluation of water quality dynamics across river systems ^25^. Remote sensing platforms (e.g., MODIS-Aqua, hyperspectral sensors) can monitor parameters such as turbidity, chlorophyll-a, dissolved oxygen, and suspended sediments ^26–28^, while GIS-based spatial interpolation techniques (e.g., IDW, Kriging, Thiessen polygons) predict water quality at unsampled sites, generating surface maps that reveal contamination hotspots ^29,30^. These methods also allow temporal comparisons, helping researchers track trends in pollution, seasonal variability, and the long-term impacts of industrial discharge and climate change ^25,31^.

While several studies have assessed Nile River water quality using traditional indices, most have been limited in spatial coverage, lacked integration of spatial analysis, and provided only descriptive evaluations with little methodological innovation. There remains a need for studies that combine robust geoinformatics tools with water quality indices to identify spatial patterns of pollution, highlight critical hotspots, and provide a decision-support framework for sustainable water management.

This study addresses this gap by assessing the impact of industrial wastewater on the Nile River in Aswan Governorate. Specifically, it applies GIS-based inverse distance weighting (IDW) to map spatial variations in physicochemical parameters and evaluates water suitability using the weighted arithmetic water quality index (WAWQI) and the canadian water quality index (CWQI). The study focuses on effluents from sugar, paper, and ferroalloy industries, categorizing water quality into good, poor, and unsuitable zones. By integrating geoinformatics with water quality indices, the research provides a more comprehensive assessment than previous localized studies and offers a decision-making framework to support wastewater management, policy enforcement (in line with Egypt’s Law 48/1982), and SDGs 6, 12, and 14. It hypothesizes that industrial discharges significantly deteriorate water quality, contributing to ecological degradation and a decline in fish catch.

## Materials and methods

### Study area

**Fig. 1:**
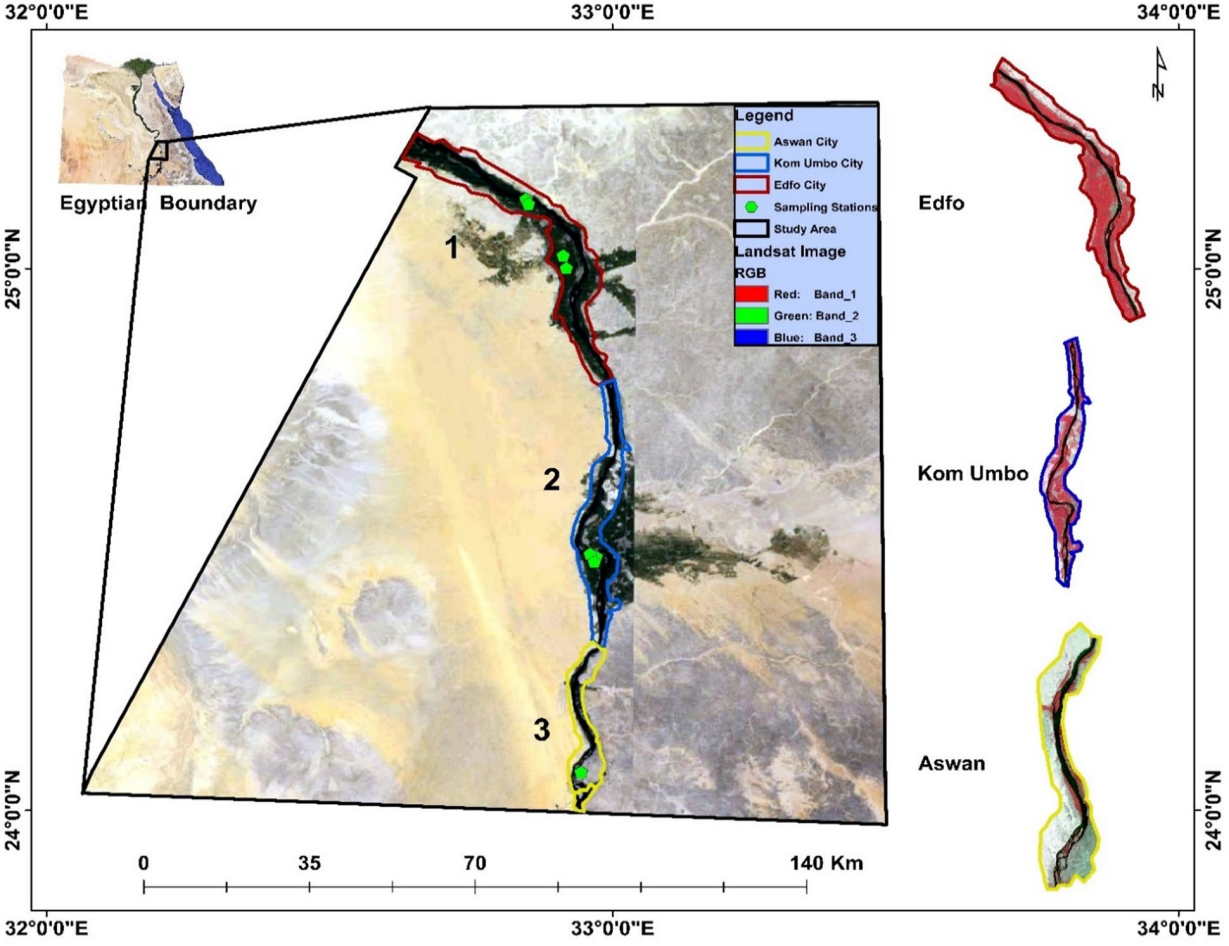
Location map of the sampling sites along the Nile River in southern Egypt. The map was created by the authors using ArcGIS 10.8 (Esri).

The study area focused on a section of the Nile River in southern Egypt, specifically along the Aswan Governorate, which stretches from Aswan City in the south to Edfu city in the north between approximately 23°58′–25°00′N latitude and 32°45′–32°55′E longitude (Fig. 1). This river stretch covers an estimated **120 km** and forms a critical freshwater corridor that supports domestic supply, agriculture, fisheries, navigation, and industrial activities. The climate of the region is hyper-arid, characterized by extremely low annual rainfall (< 1–3 mm), high evaporation rates, hot summers (often exceeding 42 °C), and mild winters. Water demands in the study area are dominated by agriculture, particularly sugar cane cultivation, followed by domestic consumption, industrial use, and fisheries. Several industries are situated directly along the riverbanks, including the Edfu Sugar Factory, Kom Ombo Sugar Factory, Misr Edfu Paper Company, and the Egyptian Ferro Alloys Company. Except for the Kom Ombo Sugar Factory which discharges via the Berba Drain these facilities release their effluents directly into the Nile River. Sugar factories operate seasonally from January to May, while the paper and ferroalloy plants operate year-round, with brief maintenance shutdowns. The region is exposed to both point sources of pollution, such as industrial wastewater outfalls, municipal sewage discharge points, and drainage canals and non-point sources, including agricultural runoff enriched with fertilizers, pesticides, and suspended sediments. Eight strategically selected sampling sites (Table 1) were distributed along the river to capture spatial variations in physicochemical characteristics and to evaluate the influence of these combined pollution sources on the local water quality.

**Table 1:** Description of the studied key sectors along the Nile River of the Aswan Governorate, South Egypt.

City	Industrial discharge source	The Nile River		Study Sites	Latitude	Longitude	Description of Water Sampling Location
		Length (Km)	Width (m)				
Aswan	Reference point with no industrial source	32.2	580–1400	S_1_	32.878	24.074	In front of the Saluja Reserve, the main channel.
					32.879	24.249	In front of the Saluja Reserve, the eastern sector of the Nile.
					32.873	24.166	In front of the Saluja Reserve, the western sector of the Nile.
Kom Ombo	Kom Ombo Sugar Factory	60.7	350–1450	S_2_	32.922	24.446	1 km upstream of Berba drain, the main channel of the Nile.
					32.925	24.735	1 km upstream Berba drain, the eastern sector of the Nile
					32.929	24.703	1 km upstream of Berba drain, the western sector of the Nile
				S_3_	32.925	24.456	In front of the Berba drain, the eastern sector of the Nile
				S_4_	32.914	24.461	3 km downstream of the Berba drain, the main channel of the Nile.
					32.908	24.329	3 km downstream of the Berba drain, the eastern sector of the Nile.
					32.913	24.342	1 km downstream of the Berba drain, the western sector of the Nile.
Edfu	Edfu Sugar Factory Edfu Paper Factory Ferroalloys Factory	56.9	550–1400	S_5_	32.886	24.988	North of Edfu Bridge, the main channel of the Nile
					32.694	25.206	North of Edfu Bridge, the eastern sector of the Nile.
					32.743	25.168	North of Edfu Bridge, the western sector of the Nile.
				S_6_	32.881	25.011	In front of the ferroalloys factory discharge, the eastern sector of the Nile
				S_7_	32.859	25.047	In front of the sugar and paper factories discharge, the western sector of the Nile
				S_8_	32.855	25.055	1 km downstream of Edfu sugar factory discharge, the main channel of the Nile
					32.891	24.847	1 km downstream of Edfu sugar factory discharge, the eastern sector of the Nile.
					32.898	24.812	1 km downstream of Edfu sugar and paper factories discharge, the western sector of the Nile

### Physicochemical analysis of water samples

In 2024, water samples were collected from eight sites (S1–S8) along the Nile River in Aswan during both the winter and summer seasons. Each site was subdivided into multiple sampling points, yielding a total of 18 points across all locations. Water samples were collected at a depth of 1 m using a Van Dorn water sampler. For each season, 54 samples were obtained, corresponding to the eight main sampling sites listed in Table 1. At each point, three samples were collected from the main channel, the eastern sector, and the western sector of the river to capture lateral variability in water quality during each season. Field physical and chemical parameters, including temperature, pH, turbidity, and dissolved oxygen (DO), were measured directly using a Horiba field multi-item water quality checker (Horiba U-10). Physicochemical parameters include: total dissolved solids (TDS), total suspended solids (TSS), chemical oxygen demand (COD), biological oxygen demand (BOD), total nitrogen (TN), nitrate (NO_3_-N), ammonia (NH_3_-N), total phosphorus (TP), sulfates (SO₄), chlorides, fluoride, alkalinity, hardness, and phenol were measured in laboratory according to standard method ^32,33^. High-grade chemicals (Merck and Sigma Aldrich) are used for chemical analysis. Field and laboratory measurements were conducted using quality control and assurance standards, such as instrument calibration, the use of blank, triplicate, control charts, and reference standards, high-quality chemicals, to guarantee accurate and trustworthy measurements.

### Water quality index (WQI)

Assessing water quality is essential to ensure the protection of public health and sustainability of aquatic ecosystem. Water quality indices (WQIs) provide an efficient method translating complex water chemistry datasets into easily interpretable scores that support environmental monitoring and management. In this study, two complementary indices the weighted arithmetic water quality index (WAWQI) and the canadian water quality index (CWQI) were selected due to their global acceptance, robustness and suitability for evaluating multi parameter freshwater systems. Evaluating water quality is essential for ensuring both public health and the well-being of aquatic ecosystems ^34,35^.

WAWQI and CWQI were selected based on several key criteria. First, both indices are widely applied internationally for surface water quality assessment, ensuring comparability with global freshwater studies. Second, they effectively integrate multiple physicochemical parameters into a single representative value, facilitating interpretation in environmental assessments. Third, they are compatible with regulatory frameworks, particularly Egyptian Law No. 48/1982 for surface water protection in the case of WAWQI, and international aquatic life protection guidelines for CWQI. In addition, both indices demonstrate strong scientific validity and sensitivity to pollution sources, allowing the detection of spatial variations, especially near industrial discharge points. Finally, their use involves well-established and reproducible computational procedures, enhancing transparency and methodological consistency.

#### Weighted arithmetic water quality index (WAWQI)

WAWQI is an enhanced form of Horton’s original model, introduced by Brown and colleagues in 1970. This index serves as a key tool for assessing and categorizing surface water quality, offering critical insights for environmental management and policy ^36^. In this study, the WAWQI method is applied to determine the suitability of the Nile River’s water based on the criteria set forth in Egyptian Law No. 48 of 1982, which governs the protection of the Nile from pollution. The WAWQI is computed using the formula^37^:

1 $$\:WAWQI=\frac{\boldsymbol{\varSigma\:}\:\boldsymbol{W}\boldsymbol{i}\:\boldsymbol{q}\boldsymbol{i}}{\boldsymbol{\varSigma\:}\boldsymbol{W}\boldsymbol{i}}$$

Where *q*_*i*_ is the quality rating for each individual parameter, and *Wi* denotes the relative weight assigned to that parameter, reflecting its significance in water quality assessment. WAWQI results are interpreted based on the following classification: Excellent (0–25), Good (26–50), Poor (51–75), Very Poor (76–100), and Unsuitable for use (> 100) ^38^.

The WAWQI was calculated by first assigning standard permissible limits (Si) for each water quality parameter according to Egyptian Law No. 48/1982. For each parameter, a quality rating (qi) was then computed using the equation:

2 $$q_{i}\:={\left(\frac{{V}_{i}\:-\:{V}_{0}}{{S\:}_{i}-\:\:{V}_{0}}\right)\times\:\:}^{100}$$

where $\:{V}_{i}$represents the measured value, $\:{V}_{0}$is the ideal value, and $\:{S}_{i}$is the standard limit. Subsequently, weights (Wi) were assigned to each parameter based on their relative importance to human and ecological health, their influence on overall water pollution, and weighting schemes commonly adopted in previous studies.

WAWQI was then calculated using Eq. (1), after which water quality was classified into five categories: excellent (0–25), good (26–50), poor (51–75), very poor (76–100), and unsuitable (> 100). Weights were assigned following established methodologies, whereby parameters associated with greater human-health or ecological risks (e.g., COD, BOD, TN, and phenol) were given higher weights, while less critical parameters such as hardness or alkalinity received lower weights. This weighting approach ensures the index better reflects real-world environmental risks and remains consistent with national guideline priorities.

#### Canadian water quality index (CWQI)

In this analysis, CWQI is employed to assess how well the Nile River meets the standards necessary for sustaining aquatic life, following the Canadian Council of Ministers of the Environment (CCME) water quality guidelines (2001). The CWQI is calculated as:

3 $$\:\boldsymbol{C}\boldsymbol{W}\boldsymbol{Q}\boldsymbol{I}=100-\left(\frac{\sqrt{{\boldsymbol{F}1}^{2}+{\boldsymbol{F}2}^{2}+{\boldsymbol{F}3}^{2}}}{1.732}\right)$$

Where F1 (Scope) represents the percentage of parameters that exceed water quality objectives, F2 (Frequency) indicates the percentage of tests that fail to meet these objectives, and F3 (Amplitude) quantifies how much the failed test results deviate from guideline values. Based on the classification system developed by the CCME ^39,40^, the water quality status is categorized as Excellent (95–100), Good (80–94), Fair (65–79), Marginal (45–64), or Poor (0–44).

### Data processing and GIS analyses

After measuring seasonal variations in water quality parameters, a geodatabase was developed to preprocess sampling data for each sector, focusing on the impact of industrial discharge. The collected data were then spatially interpolated using the inverse distance weighting (IDW) method in ArcGIS 10.8 to generate continuous water quality maps for each Nile sector. It assumes that samples closer to the prediction location have more influence than those farther away, which aligns with the localized nature of industrial discharge effects on water quality. IDW was chosen for its computational efficiency, ease of implementation, and ability to handle moderately sparse environmental datasets, where localized effects of industrial discharge dominate. Unlike more complex geostatistical approaches such as kriging, IDW does not require variogram modeling, making it particularly suitable for datasets with limited spatial continuity analysis. To ensure reliability, IDW outputs were validated against observed data through cross-validation, where root mean square error (RMSE) and mean absolute error (MAE) were calculated. Results showed that IDW produced acceptable prediction accuracy with relatively low error values, supporting its suitability for this study. Additionally, IDW’s strong integration with ArcGIS facilitated rapid generation of interpolated surfaces, enabling efficient spatial visualization of multiple parameters. Additionally, indices such as the CWQI and the WAWQI were represented geographically on separate maps.

Environmental parameters such as TDS, alkalinity, BOD, COD, fluoride, hardness, NH₃-N, NO₃-N, SO₄, TN, TP, TSS, Cl, phenol, temperature, DO, conductivity, turbidity, and pH were each mapped individually. These spatial visualizations provided insights into the relationships between environmental conditions and their geographic distribution across sampling sites. To estimate values at unsampled locations, the IDW equation was applied as follows:

4 $$\:\boldsymbol{z}\boldsymbol{j}=\:{\sum\:}_{\boldsymbol{i}}\frac{{\boldsymbol{z}}_{\boldsymbol{i}}}{{\boldsymbol{d}}^{\boldsymbol{n}}\:\boldsymbol{i}\boldsymbol{j}}\:\div{\sum\:}_{\boldsymbol{i}}\frac{1}{{\boldsymbol{d}}^{\boldsymbol{n}\:\:}\:\boldsymbol{i}\boldsymbol{j}}$$

Where *z*_*i*_ represents the observed value at a known location, *d*_*i*j_ is the distance to the known point, *z*_*j*_ is the estimated value at the unknown location, and *n* is a user-defined exponent controlling the influence of nearby points.

### Validation of interpolation

To evaluate the predictive performance of IDW method, a leave-one-out cross-validation (LOOCV) approach was applied. In this procedure, each observed value at a sampling site was sequentially omitted from the dataset, and its value was re-estimated using the remaining sampling points. The prediction accuracy was then quantified by comparing the estimated values against the observed ones. Two statistical error metrics were used:

5 $$\:\boldsymbol{R}\boldsymbol{M}\boldsymbol{S}\boldsymbol{E}=\sqrt{\frac{1}{\boldsymbol{n}}\sum\:_{\boldsymbol{i}=1}^{\boldsymbol{n}}{\left({\boldsymbol{z}}_{\boldsymbol{i}-{\widehat{\boldsymbol{Z}}}_{\boldsymbol{i}}}\right)}^{2}}$$

6 $$\:\boldsymbol{M}\boldsymbol{A}\boldsymbol{E}=\frac{1}{\boldsymbol{n}}\sum\:_{\boldsymbol{i}=1}^{\boldsymbol{n}}\left|{\boldsymbol{z}}_{\boldsymbol{i}-{\widehat{\boldsymbol{Z}}}_{\boldsymbol{i}}}\right|$$

where ***Z***_***i​***_ is the observed value at location *i*, $\:{\widehat{\boldsymbol{Z}}}_{\boldsymbol{i}\:\:}$is the predicted value from IDW, and *n* is the number of sampling points. RMSE provides a measure of the overall magnitude of prediction error, while MAE offers a robust indicator of average deviation, less sensitive to extreme outliers. Together, these indices enabled an objective assessment of the reliability and accuracy of the interpolated water quality surfaces, ensuring that the generated spatial patterns adequately represented field conditions.

### Statistical analysis

To better understand the dynamics of pollutants in the Nile River, the linear relationship between the variables (physicochemical parameters, WAWQI, and CWQI) was confirmed using Pearson’s correlation coefficients. The Excel PEARSON Function (Microsoft Excel) was used to compute Pearson’s correlation coefficient. The linearity assumption for correlation was tested, and the person correlation coefficient was computed.

## Results and discussion

**Fig. 2:**
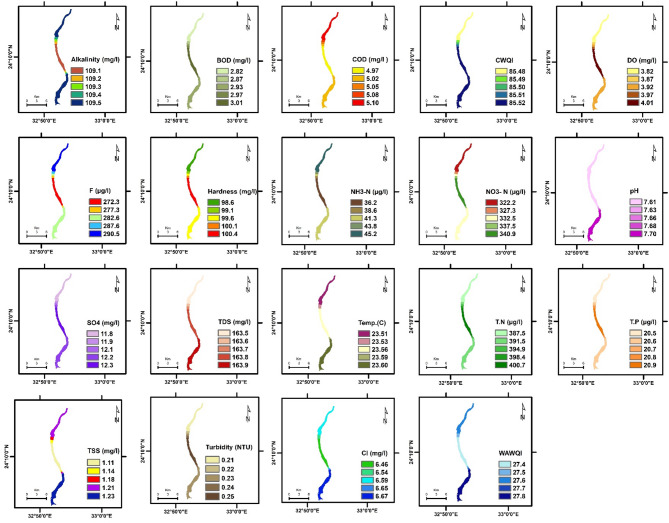
The spatial distribution of water quality parameters in Aswan Nile River (Summer).

**Fig. 3:**
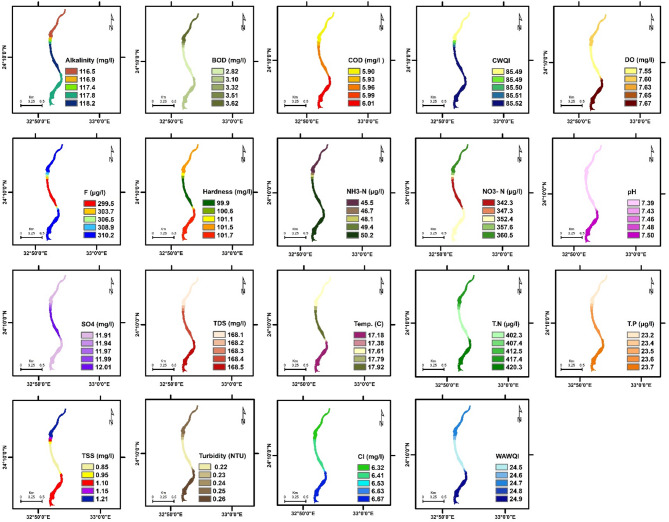
The spatial distribution of water quality parameters in Aswan Nile River (Winter).

**Fig. 4:**
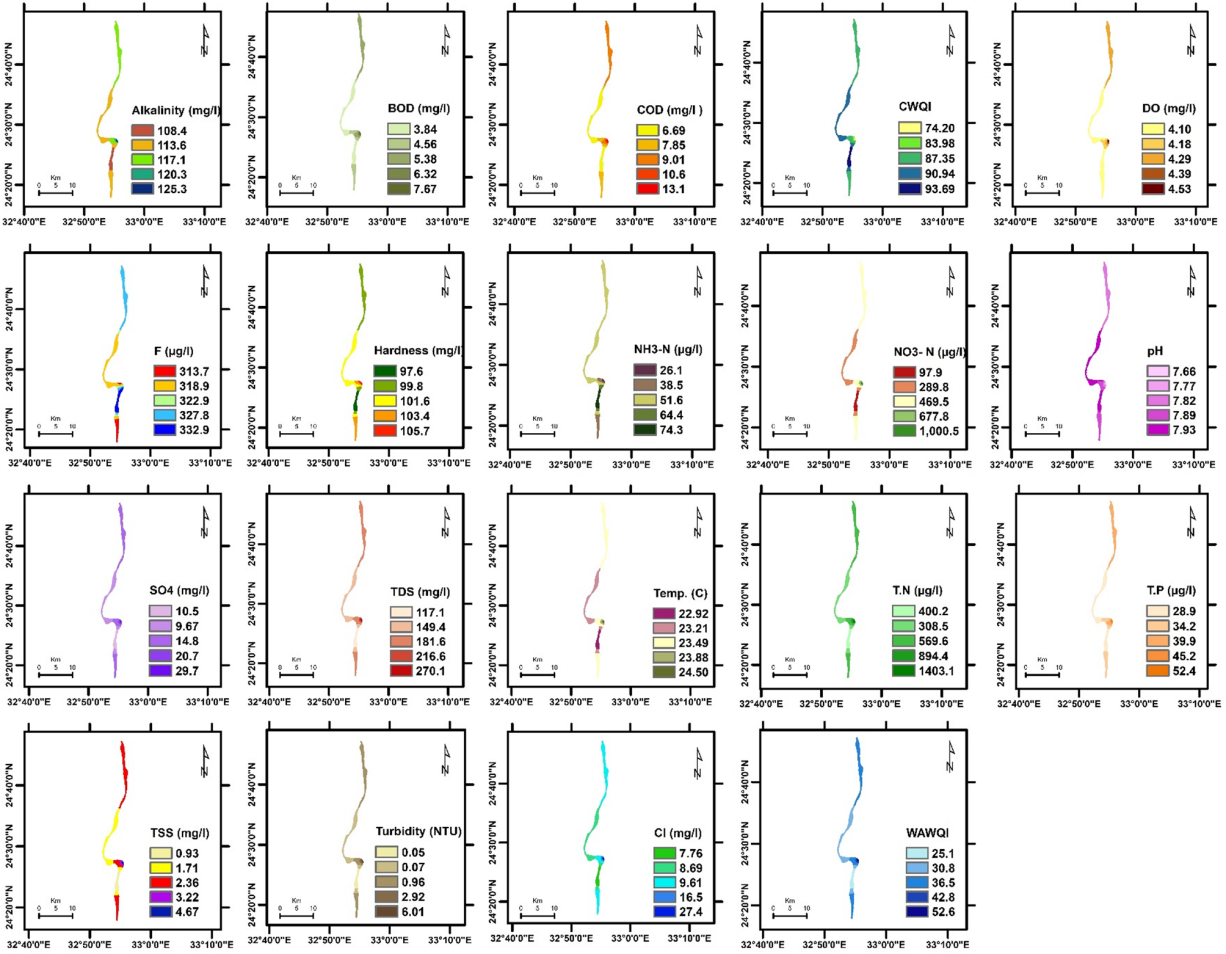
The spatial distribution of water quality parameters in Kom Ombo Nile River (summer).

**Fig. 5:**
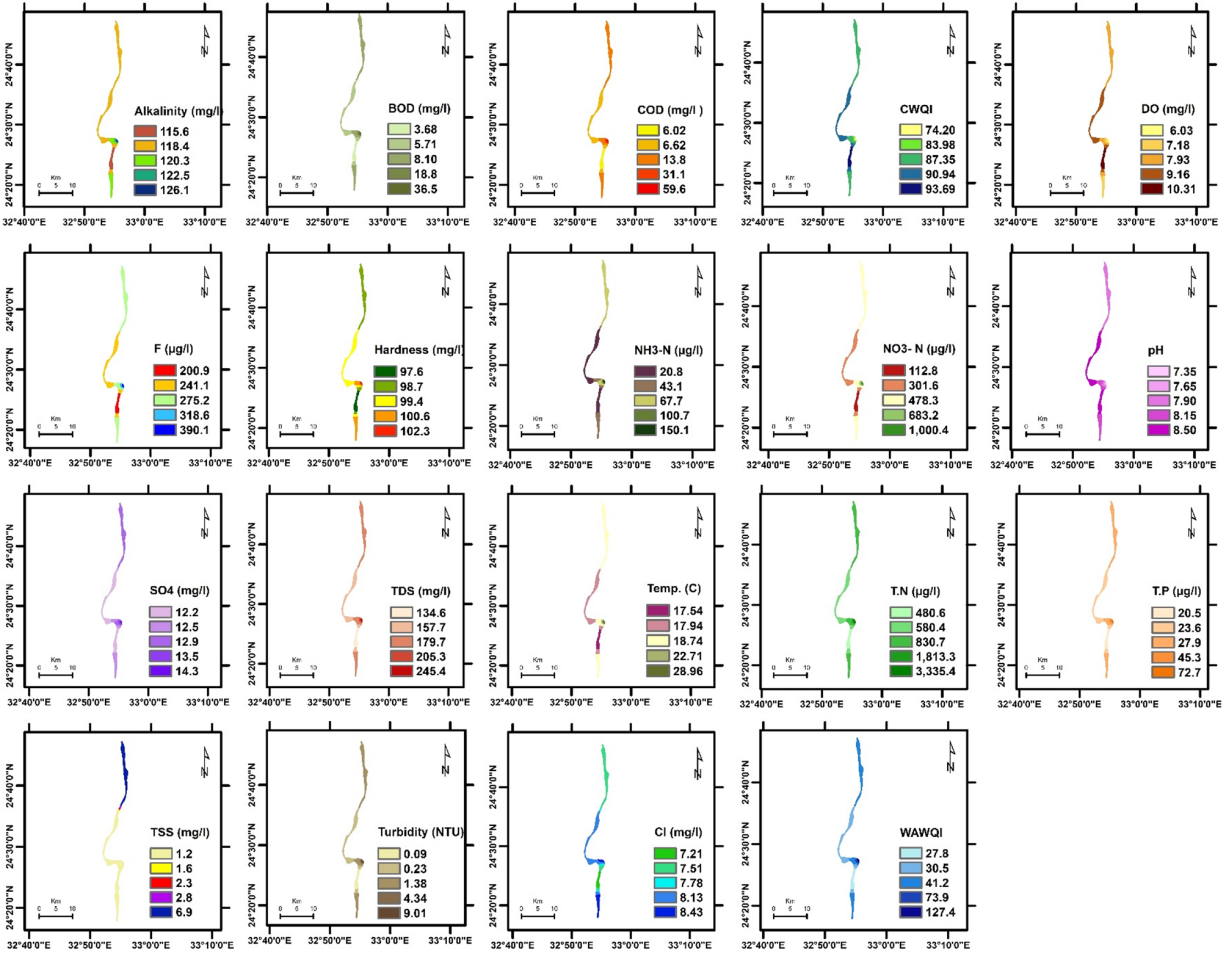
The spatial distribution of water quality parameters in Kom Ombo Nile River (winter).

**Fig. 6:**
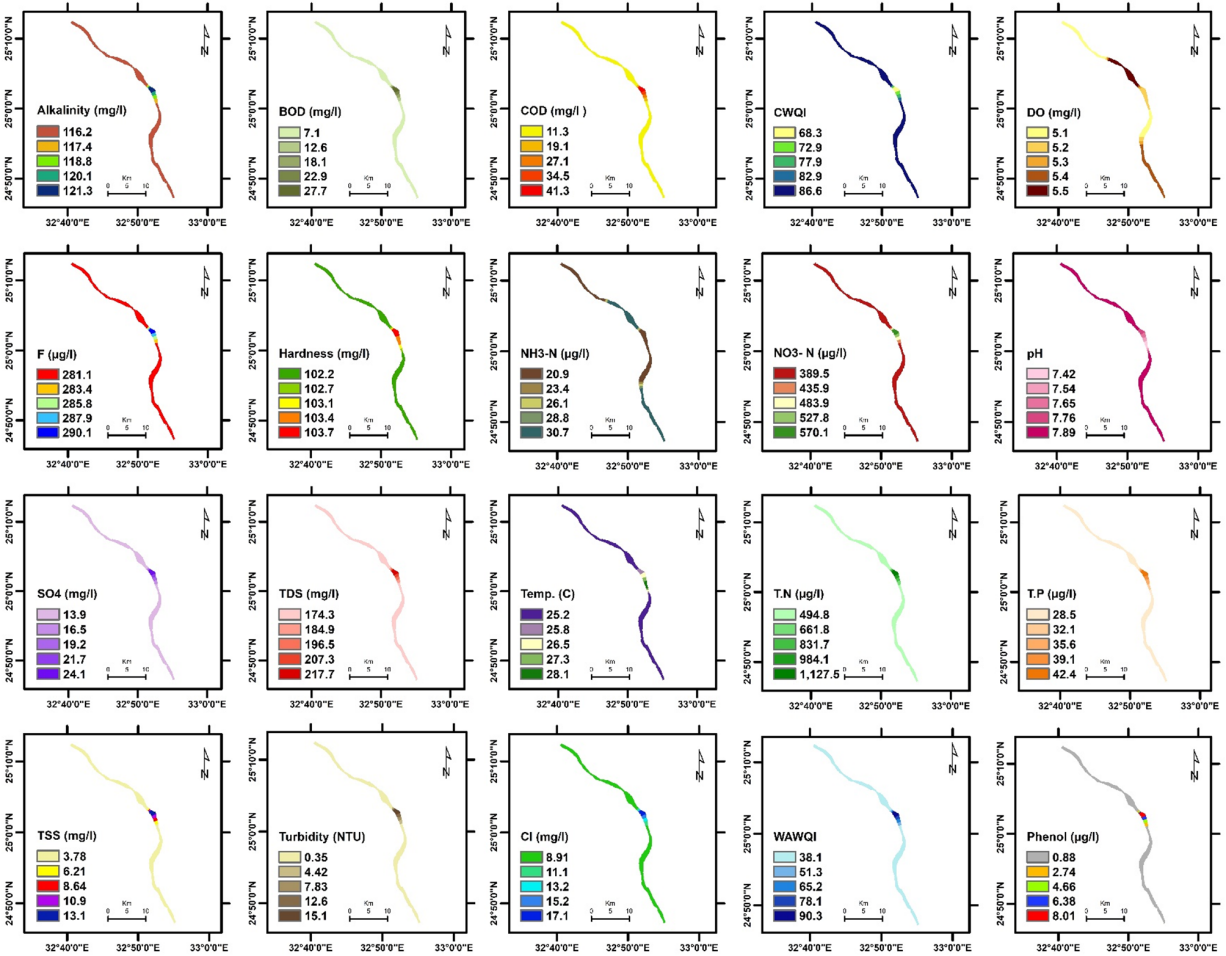
The spatial distribution of water quality parameters in Edfu Nile River (summer).

**Fig. 7:**
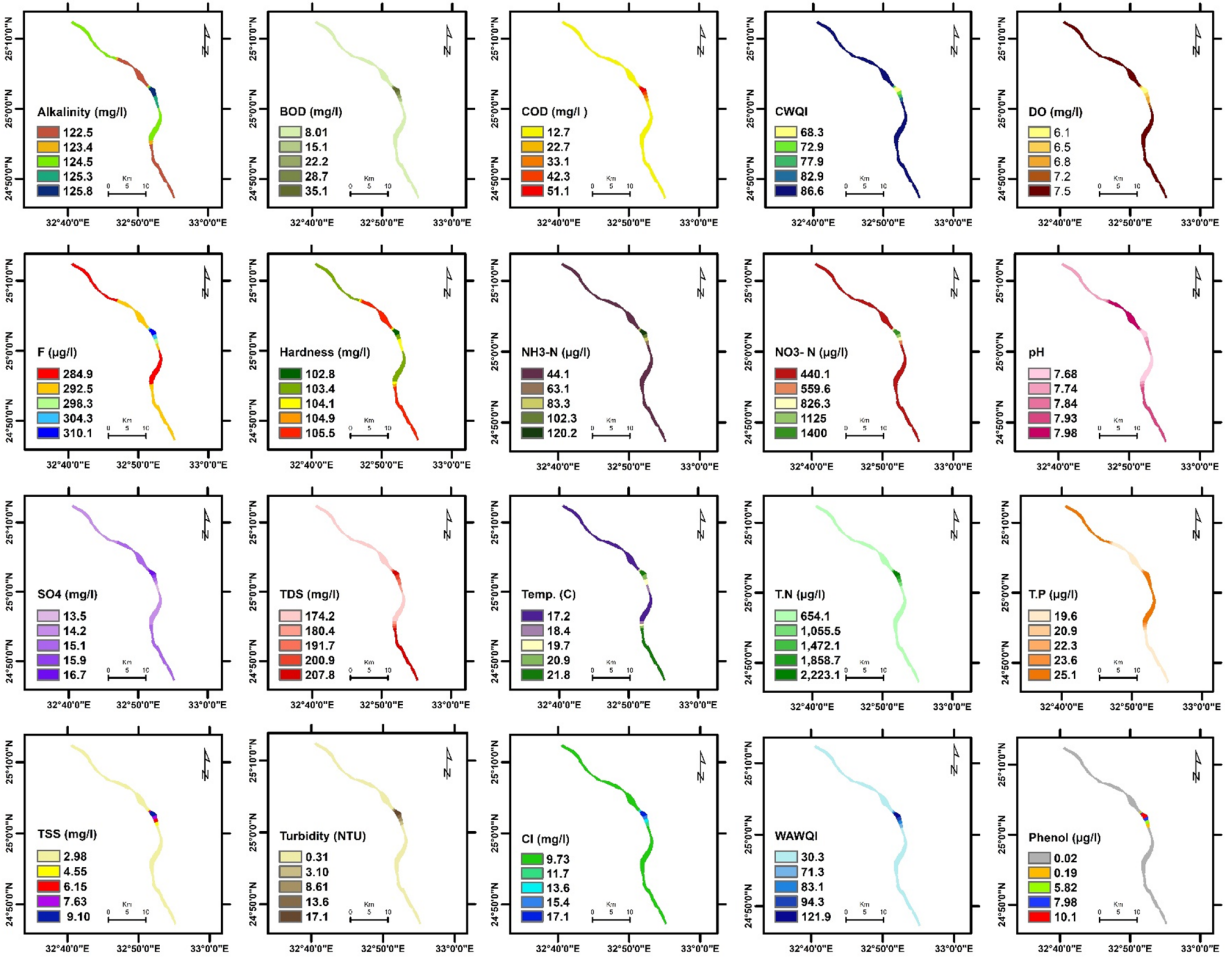
The spatial distribution of water quality parameters in Edfu Nile River (winter).

The spatial distribution of physicochemical parameters in the Nile River along Aswan Governorate reveals seasonal variations between summer and winter, reflecting changes in environmental conditions and potential pollutant sources. These variations provide insight into water quality dynamics and their potential influence on aquatic ecosystems. Key findings are summarized below, followed by a comprehensive discussion of their ecological implications. The detailed seasonal patterns and values are presented in Figs. 2, 3, 4, 5, 6 and 7.

### Validation of interpolations

To evaluate the accuracy of the IDW interpolations shown in Figs. 2, 3, 4, 5, 6 and 7 and a leave-one-out cross-validation (LOOCV) procedure was applied for all measured parameters. For each run, one sampling point was omitted, and its value was estimated using the remaining dataset. The differences between observed and predicted values were quantified using RMSE and MAE.

The validation results indicated that interpolation errors were generally low for relatively stable parameters such as pH (RMSE = 0.08, MAE = 0.06), temperature (RMSE = 0.4 °C, MAE = 0.3 °C), and alkalinity (RMSE = 4.5 mg/L, MAE = 3.2 mg/L). Moderate errors were observed for parameters influenced by both natural and anthropogenic variability, including DO (RMSE = 0.6 mg/L, MAE = 0.4 mg/L), TDS (RMSE = 12.3 mg/L, MAE = 9.1 mg/L), and chloride (RMSE = 0.5 mg/L, MAE = 0.4 mg/L). Higher errors were associated with parameters strongly affected by localized industrial discharges, such as TSS (RMSE = 1.1 mg/L, MAE = 0.8 mg/L), turbidity (RMSE = 0.9 NTU, MAE = 0.7 NTU), and NH₃-N (RMSE = 12.8 µg/L, MAE = 9.5 µg/L) as showing in Table 2.

**Table 2:** Results of leave-one-out cross-validation (LOOCV) for IDW interpolations of water quality parameters in the Nile River (Aswan Governorate). Validation was assessed using root mean square error (RMSE) and mean absolute error (MAE), indicating acceptable prediction accuracy across most parameters.

Parameter	RMSE	MAE	Unit
pH	0.08	0.06	–
Temperature	0.40	0.30	°C
Alkalinity	4.50	3.20	mg/L
Hardness	3.60	2.70	mg/L
DO	0.60	0.40	mg/L
TDS	12.30	9.10	mg/L
Chloride (Cl)	0.50	0.40	mg/L
SO₄	1.90	1.40	mg/L
BOD	1.20	0.90	mg/L
COD	2.80	2.10	mg/L
NH₃-N	12.80	9.50	µg/L
NO₃-N	15.40	11.20	µg/L
TN	25.50	18.90	µg/L
TP	6.20	4.70	µg/L
Phenol	0.90	0.70	µg/L
TSS	1.10	0.80	mg/L
Turbidity	0.90	0.70	NTU
Fluoride (F)	8.10	6.20	µg/L
CWQI	2.40	1.90	Index
WAWQI	3.10	2.30	Index

Overall, the cross-validation confirmed that the IDW method provided robust and reliable spatial predictions, with prediction errors remaining within acceptable limits for environmental assessment. The higher uncertainty observed in localized pollutants underscores the importance of dense sampling in areas near industrial outfalls to further improve spatial accuracy.

### Organic matter and related parameters

High COD and BOD values in water bodies indicate a significant organic matter load, likely due to pollution in aquatic ecosystems. This affects water quality and can lead to algal blooms, impacting the environment, human health, and the economy ^11,41^. In summer, COD and BOD values at the mainstream of the Nile River varied from 4.97 to 11.3 mg/L, 2.82 to 7.1 mg/L, respectively. The highest COD and BOD values were exhibited near the industrial discharge point (sugar and paper factories), where COD and BOD values ranged from 13.1 to 41.3 mg/L and 7.67 to 27.7 mg/L, respectively. In winter, the values of COD and BOD were generally higher compared to summer. In the mainstream, COD and BOD levels ranged from 5.9 to 12.7 mg/L and 2.82 to 8.01 mg/L, respectively. Near the industrial discharge point, COD and BOD values were significantly higher, ranging from 51.1 to 59.6 mg/L and 35.1 to 36.5 mg/L, respectively. Additionally, the results show that the COD and BOD levels in the mainstream in Aswan and Kom Ombo ranged between 4.97 and 6.69 mg/L and 2.82 and 3.84 mg/L, respectively. These values fall within the acceptable limits set by Egyptian law (≤ 10 mg/L). They were, nevertheless, above the allowed limits in Edfu City, which were between 11.30 and 12.70 mg/L and 7.10 and 8.01 mg/L, respectively. The BOD to COD ratio was above 0.5 during summer and winter, ranging from 0.50 to 0.70. This indicates the high biodegradability of the monitored organic pollutants ^42^.

Dissolved oxygen levels have a significant impact on the health of aquatic organisms, supporting a variety of species and increasing resilience to environmental changes. It is a key water quality parameter and a direct indicator of pollution ^43,44^. The concentration of DO varies seasonally. In the summer, DO levels ranged from 3.82 to 5.50 mg/L, which is lower than the winter range of 6.03 to 10.31 mg/L. In the summer, dissolved oxygen levels are all below the legal limits set by Egypt (≥ 6), but in the winter, they are all within acceptable bounds. The decrease in dissolved oxygen values in the summer is due to the High Dam releasing deep, low-oxygen water into the Nile ^45^. Additionally, high summer temperatures in southern Egypt, exceeding 45 °C, hinder the efficient dissolution of oxygen. Findings from Qena Governorate indicate that seasonal variations in temperature and flow significantly influence dissolved oxygen and chemical concentrations, posing potential risks to aquatic ecosystems and limiting water usability ^25^. These findings emphasize the need for targeted and sustainable water management strategies to address increasing environmental pressures.

Phenolic compounds are significant environmental contaminants resulting from both natural and human activities. Human sources, such as chemical plants, oil refineries, and paper mills, contribute larger quantities of these compounds ^46^. They are highly toxic, even at low concentrations ^47^. Due to their non-biodegradable nature, phenolic compounds persist in aquatic systems for extended periods ^48^. When concentrations exceed 1 mg/L, phenols can cause serious harm to aquatic life ^49^. High concentrations of phenol were detected only near paper mill discharges in the Edfu sector of the Nile River, ranging from 8.01 to 10.1 µg/L, which exceeded Egyptian legal limits (1 µg/L).

A strong positive correlation was observed between COD and several key parameters, including BOD (*r* = 0.99, *p* < 0.05), Total Nitrogen (*r* = 0.92, *p* < 0.05), and NH₃ (*r* = 0.75, *p* < 0.05), indicating a close relationship among these indicators of organic and nutrient pollution. In contrast, DO exhibited negative correlations with COD (*r* = −0.30), BOD (*r* = −0.29), and NO₃ (*r* = −0.24, *p* < 0.05), suggesting that increased pollutant loads may contribute to reduced oxygen levels in the water (Table 3). This indicates that there is oxygen consumption occurring during biodegradation and nitrification processes at Nile River sites that have a significant organic load ^50^. The strong relation between COD and BOD directly reflects ecosystem health and potential stress on aquatic life, while the correlation between COD and NH_3_ relates more to toxicity risks.

**Table 3:** Pearson correlation coefficient (r-value) between measured physicochemical parameters, P-value < 0.05.

	Temp	*p* H	TDS	DO	Turb.	COD	BOD	T.N	T.P	SO4	NO_3_- N	F	NH_3_-N	TSS	Cl	Alk.	Hard.	Phenol	WAWQI	CMWQI
Temp	1.000																			
pH	−0.545	1.000																		
TDS	0.382	−0.453	1.000																	
DO	−0.876	0.331	−0.324	1.000																
Turbidity	0.336	−0.335	0.665	−0.238	1.000															
COD	0.417	−0.573	0.650	−0.308	0.894	1.000														
BOD	0.404	−0.541	0.634	−0.296	0.915	0.998	1.000													
T. N	0.446	−0.703	0.773	−0.372	0.744	0.920	0.901	1.000												
T. P	0.664	−0.666	0.792	−0.603	0.435	0.625	0.587	0.760	1.000											
SO_4_	0.251	−0.053	0.833	−0.213	0.585	0.339	0.347	0.355	0.485	1.000										
NO_3_- N	0.266	−0.426	0.769	−0.240	0.819	0.794	0.801	0.857	0.497	0.533	1.000									
F	0.473	−0.674	0.602	−0.528	0.381	0.625	0.590	0.776	0.831	0.200	0.550	1.000								
NH_3_-N	0.131	−0.617	0.315	−0.122	0.483	0.753	0.737	0.812	0.406	−0.178	0.639	0.641	1.000							
TSS	0.386	−0.249	0.627	−0.252	0.948	0.837	0.856	0.630	0.469	0.619	0.636	0.300	0.306	1.000						
Cl	0.197	−0.032	0.813	−0.172	0.628	0.360	0.376	0.398	0.385	0.954	0.678	0.171	−0.090	0.587	1.000					
Alkalinity	−0.167	−0.265	0.664	0.293	0.530	0.558	0.552	0.610	0.373	0.470	0.668	0.335	0.386	0.473	0.538	1.000				
hardness	0.095	−0.049	0.476	0.019	0.252	0.151	0.150	0.179	0.241	0.542	0.285	0.180	−0.240	0.300	0.544	0.613	1.000			
Phenol	0.117	−0.037	0.305	−0.038	0.895	0.681	0.725	0.416	0.022	0.395	0.612	0.037	0.300	0.850	0.472	0.333	0.128	1.000		
WAWQI	0.431	−0.556	0.694	−0.345	0.908	0.993	0.993	0.937	0.633	0.390	0.854	0.640	0.748	0.830	0.432	0.571	0.174	0.688	1.000	
CMWQI	−0.352	0.317	−0.729	0.258	−0.991	−0.857	−0.877	−0.724	−0.476	−0.685	−0.813	−0.378	−0.403	−0.952	−0.713	−0.539	−0.302	−0.864	−0.877	1.000

### Nutrient pollution (Eutrophication risk)

Exposure of Nile water to nitrogenous pollutants increases the risk of eutrophication in the river. This process can lead to toxic algal blooms, depletion of oxygen, and a decline in biodiversity in the Nile River ^51–53^. Total nitrogen values ranged from 387.5 to 3345.4 µg/L, which falls within the permissible limit of 3500 µg/L according to Egyptian standards. However, total nitrogen concentrations were significantly higher only near the discharge points of sugar and paper factories, with levels ranging from 1127.5 to 3345.4 µg/L. In contrast, total nitrogen levels in most parts of the Nile River ranged from 387.5 to 654.1 µg/L, as some of the fertilizers used in agriculture are drained into the river through agricultural drains ^54^. Nitrate concentrations ranged from 97.5 to 440 µg/L in most sections of the Nile River during the study periods, which is within the normal limits. However, these levels increased near industrial pollution sources, where they ranged from 570.1 to 1400 µg/L. This may be due to the biodegradation of industrial organic matter and biological nitrification of ammonia ^55^. More than 1 mg/L ammonium and nitrogen concentrations indicate organic pollution, they can be harmful to aquatic life if they exceed 2.5 mg/L ^56^. Low concentrations of ammonia-nitrogen (NH_3_-N) were observed during the summer and winter, ranging from 20.8 to 150.1 µg/L, which is within the permissible limits of Egyptian law (< 500 µg/L).

Total phosphorus concentrations ranged from 19.6 µg/L to 72.7 µg/L during both winter and summer, which still do not exceed the recommended limit of 2 mg/L in the River Nile of Egypt. This may be attributed to a lack of direct sources of pollutants with high phosphorus concentrations. Elevated phosphorus levels can encourage algae growth, negatively affecting water quality for municipal, recreational, and fisheries use. To address eutrophication, the USEPA recommends a limit of 0.1 mg/L for total phosphorus in flowing waters ^57^.

### General physicochemical characteristics

The water temperature varied significantly between summer and winter. In summer, it ranged from 22.92 to 28.10 °C. In winter, the water temperature in most sections of the Nile River ranged from 17.20 °C to 17.92 °C, while it exceeded 21 °C only near the discharge points of sugar and paper factories. This is due to the discharge of cooling water with a high heat load. The water temperature of the Nile River in all study areas was within a suitable range for fish and aquatic organisms ^25^. But the summer season is more suitable than winter, where in the summer season, when the water temperature exceeds 22 °C, it is most suitable for Nile fish, especially Nile tilapia, in terms of optimum growth performance and survival ^58,59^. The pH values ranged from 7.35 to 7.98 during both summer and winter. These values fall within the acceptable range of 6.5 to 8.5 for freshwater, as defined by Egyptian Law No. 48/1982 ^60^, and meet the freshwater criteria for aquatic life established by the CCME ^61^. TDS concentrations in both winter and summer were within Egypt’s legal limits (less than 500 mg/L), ranging from 134.6 to 270.1 mg/L.

TSS levels in most sectors of the Nile River ranged from 0.85 to 3.78 mg/L during both the winter and summer seasons. TSS levels near industrial waste sources have increased significantly, reaching levels between 4.67 and 13.1 mg/L, reducing dissolved oxygen and raising temperatures, potentially affecting the survival of small fish ^62^. Turbidity affects the levels of dissolved oxygen in water columns, which in turn influences the rates of photosynthesis ^63^. Industrial discharges have impacted the transparency of the Nile River’s water near sugar and paper factories, resulting in turbidity levels ranging from 6.01 to 17.10 NTU. In contrast, undisturbed areas exhibit low turbidity levels, which were lower than 1 NTU during both winter and summer seasons. The northern section of the Nile River in Qena Governorate exhibits higher conductivity and pH levels, likely indicating pollutant accumulation due to reduced flow ^25^. In contrast, the central and southern regions are characterized by elevated TDS and turbidity, primarily attributed to localized anthropogenic activities.

Alkalinity is crucial for aquatic life as it buffers against pH changes. Freshwater pH stabilizes when total alkalinity is 100–200 mg/L but drops below 10 mg/L make the system susceptible to pH variations ^64^. The results indicated that the alkalinity and hardness concentrations were within acceptable limits, especially for aquatic organisms, with values recorded between 108.4 and 126.1 mg/L for alkalinity and 97.3 to 105.7 mg/L for hardness during both winter and summer. Additionally, the findings confirmed that industrial wastewater did not significantly affect the total alkalinity and hardness of the Nile River. Normal freshwater chloride concentrations typically range from 1 to 100 mg/L, and levels should not exceed 250 mg/L ^61,65^. In this study, chloride concentrations were found to be within the normal range, measuring between 6.32 mg/L and 27.4 mg/L during winter and summer, where, in accordance with CCME guidelines, the appropriate chloride levels for aquatic life do not surpass 120 mg/L.

Fluoride toxicity in aquatic invertebrates and fish increases with fluoride concentration, with safe levels below 500 µg F^−^/L recommended to protect freshwater animals from fluoride pollution ^66^. The fluoride levels in the Nile River ranged from 200.9 to 332.9 µg/L, which is below the acceptable limits set by Egyptian law, which are 500 µg/L for fluoride. High sulfate concentrations can enhance the mobilization of phosphorus in river sediments and wetlands ^67,68^, which may lead to eutrophication in surface waters. In the study area, sulfate levels were below the acceptable limit set by Egyptian law, which is 200 mg/L, ranging from 10.5 to 29.7 mg/L.

### Water quality indices

WQI is crucial for assessing water quality, protecting aquatic life, and ensuring the safety of agricultural and drinking water. It offers a single rating that helps in choosing suitable treatment methods ^69,70^. The current study employed WAWQI and CWQI to assess the water quality of the Nile River. The WAWQI assessed if the Nile River water meets Egyptian Law’s criteria (No. 48/1982) for drinking, irrigation, industry, and fish production, while the CWQI assessed if the water meets aquatic life protection standards. During the summer and winter, the WAWQI values along the Nile River in the Aswan governorate varied. In summer, the value ranged from 25.1 (good quality) to 38.1 (good quality), while in winter, they ranged from 24.5 (excellent quality) to 30.3 (good quality). However, near industrial pollution sources, the WAWQI values were significantly higher, ranging from 52.6 (poor quality) to 127.4 (unsuitable quality) during both winter and summer.

The CWQI values in most parts of the Nile River, particularly in areas distant from industrial discharge sites—ranged from 93.69 to 85.48, indicating generally good water quality and a supportive environment for aquatic life. However, CWQI values were noticeably lower near zones affected by industrial activities, ranging between 74.2 and 68.3, which reflects fair water quality and suggests localized ecological degradation. A strong positive correlation was observed between the Weighted WAWQI and parameters such as COD (*r* = 0.99, *p* < 0.05), BOD (*r* = 0.99, *p* < 0.05), turbidity (*r* = 0.90, *p* < 0.05), and Total Nitrogen (TN) (*r* = 0.93, *p* < 0.05). In contrast, WAWQI showed a significant negative correlation with DO (*r* = −0.34, *p* < 0.05) and pH (*r* = −0.55, *p* < 0.05). On the other hand, CWQI was strongly and negatively correlated with COD (*r* = −0.85, *p* < 0.05), BOD (*r* = −0.87, *p* < 0.05), turbidity (*r* = −0.99, *p* < 0.05), and TSS (*r* = −0.95, *p* < 0.05), indicating that these parameters are key contributors to water quality decline as assessed by the CWQI (Table 3).

The previous findings indicate that industrial drainage has significantly deteriorated the quality of Nile River water in areas near the points of industrial discharge. However, this impact is not as evident in the main course of the Nile. Several factors contribute to this, including the distance between the sources of industrial drainage in Kom Ombo and Edfu, as well as the swift current of the Nile, particularly during the flood season, which enables the river to self-purify of pollutants. Where the mean flow velocity ranged from 0.35 to 1 m/s, and the flow discharge downstream of the Aswan High Dam (AHD) varies from 80 million m^3^/day in winter to 240 million m^3^/day in summer ^71^. Furthermore, the effect of industrial waste on the Nile River is more pronounced in winter than in summer. This is primarily due to the lower water levels of the Nile during winter compared to summer, as well as the fact that industrial waste from sugar factories is typically halted during the summer months. The quality of the Nile River water near the ferroalloy factory drainage is good, as the drainage consists only of low-heat-load cooling water and is free from chemical pollutants. Examined the physicochemical properties of 28 sites along the Nile River and reported that the southern regions showed greater water transparency and lower nutrient concentrations, reflecting reduced eutrophication and minimal environmental disturbance ^72^. These observations are consistent with our findings in areas distant from industrial pollution sources. Assessed water quality at 13 sites along the southern Nile River, from Aswan to Qena, and reported an aquatic WQI ranging from 55 (marginal) to 27 (poor) ^73^. In contrast, our study found that the CWQI generally indicated good water quality across most sections of the Nile River, with only fair ratings observed near industrial hotspot areas.

Industrial effluents from sugar, paper, and allied factories have created distinct pollution plumes characterized by high COD/BOD, nutrients, TSS, turbidity, and phenols. Yet, the main Nile stem displays rapid self-purification owing to high flow (80–240 × 10⁶ m³ day⁻¹) and velocities up to 1 m s⁻¹ ^18,71^. Seasonal contrasts show greater impairment in winter, when lower river stage and factory operation coincide, compared with summer, when sugar-mill outputs cease and dilution increases. Downstream of the ferro-alloy plant, cooling-water discharge exerted minimal chemical impact, producing WQI scores classed as “good.” These longitudinal patterns agree with regional studies documenting higher transparency and lower nutrients in southern Nile reaches and contrast with more degraded northern sectors ^72,73^. Overall, while background reaches remain healthy, localized industrial drainage threatens aquatic life and renders water unsuitable for domestic and irrigation uses adjacent to outfalls. Targeted effluent treatment, year-round monitoring, and enforcement of Law 48/1982 are therefore essential to safeguard the ecological integrity of the Aswan Nile corridor.

To safeguard the Nile River’s aquatic environment and support sustainable development goals (SDG6), this study recommends implementing sustainable industrial water management practices, including advanced treatment and water reuse. Drinking water stations located near industrial discharge points should be relocated or upgraded. Continuous, real-time environmental monitoring of the river and pollution sources is essential. Additionally, further research is needed to explore other factors contributing to the decline in fish populations beyond water pollution.

## Conclusion

The Nile River in Aswan Governorate is a vital resource for drinking water, irrigation, fisheries, and tourism but remains under significant pressure from untreated industrial effluents and agricultural runoff. Using GIS-based spatial interpolation and two water quality indices (WAWQI and CWQI), this study revealed clear spatial and seasonal patterns of degradation. While areas distant from pollution sources maintained good water quality due to the river’s natural flow and self-purification, hotspots near industrial discharge points in Kom Ombo and Edfu exhibited poor to unsuitable conditions, with high organic matter, nitrogen, suspended solids, turbidity, and phenol levels. Seasonal analysis further indicated that winter posed greater pollution risks, likely due to reduced flow and sustained industrial activity. These findings emphasize the urgent need to stop the direct release of untreated wastewater and to enforce stricter and more sustainable wastewater management practices. Nevertheless, the study is limited by its reliance on physicochemical parameters alone, with relatively constrained spatial and temporal sampling. The absence of biological or ecotoxicological indicators also restricts a full understanding of ecosystem-level impacts. Future research should integrate biological monitoring, ecotoxicological testing, hydrodynamic modeling, and remote sensing–based time-series analysis to provide a more holistic and predictive assessment of river health. This work underscores the value of geoinformatics and spatially explicit water quality assessment for identifying priority areas for intervention. By doing so, it contributes to national regulatory enforcement and supports the achievement of sustainable development goals, particularly SDGs 6 (Clean Water and Sanitation), 12 (Responsible Consumption and Production), and 14 (Life Below Water).

## Supplementary Information

Below is the link to the electronic supplementary material.

Supplementary Material 1

## Data Availability

The datasets generated and analyzed during the current study are available from the corresponding author upon reasonable request.
