# Supplementary material for: Assessment of industrial pollution and water quality in the Nile River using GIS-based indices at Aswan, Egypt
Source: Sci Rep. 2025 Dec 31;16:3731. doi: 10.1038/s41598-025-33738-5 (PMC12852749; doi:10.1038/s41598-025-33738-5)
Supplement: Supplementary file 1 — Supplementary Material 1 [file 41598_2025_33738_MOESM1_ESM.docx]

**Assessment of Industrial Pollution and Water Quality in the Nile River Using GIS-Based Indices at Aswan, Egypt**

Ahmed N. A. Abdou^1^, Mohamed Hamed^2,3^*****, Abdelmonsef M. M. Hassan^4^, Mostafa A. Khaled^5^

^1^ Aquatic Ecology Department, Faculty of Fish and Fisheries Technology, Aswan University, Aswan, Egypt.

^2^ Department of Zoology, Faculty of Science, Al-Azhar University (Assiut Branch), 71524 Assiut, Egypt.

^3^ Department of Comparative Biomedical Sciences, School of Veterinary Medicine, Louisiana State University, Skip Bertman Drive, Baton Rouge, LA 70803, USA.

^4^ Egyptian Environmental Affairs Agency (EEAA), Aswan, Egypt.

^5^ Marine Science Department, National Authority for Remote Sensing &Space Sciences (NARSS), Cairo, Egypt.

***Corresponding author**: Mohamed Hamed

**Email**: [mali10@lsu.edu](mailto:mali10@lsu.edu); [mohamedhamedsayed239@gmail.com](mailto:mohamedhamedsayed239@gmail.com)


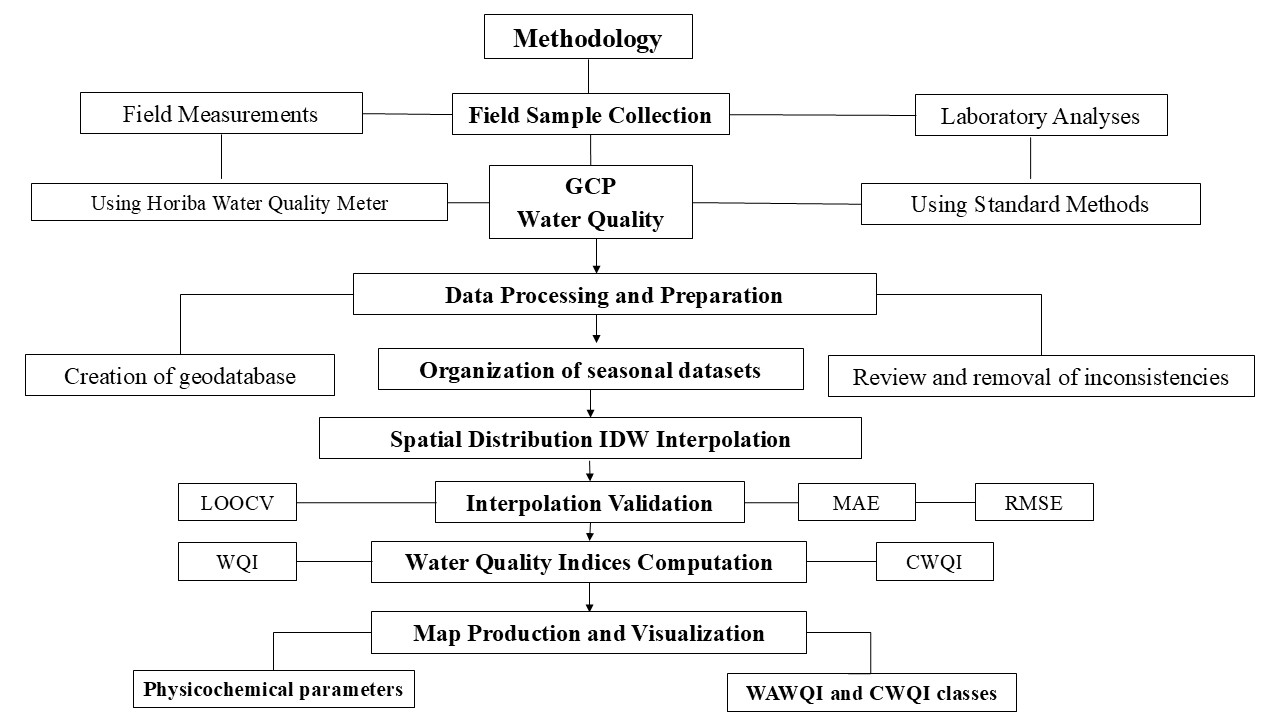


SM Fig 1. methodological workflow of the study from field sampling and laboratory analyses to GIS-based interpolation, water quality computation, validation and spatial visualization.

**SM Table 1:** Maximum and minimum mean values of the physicochemical characteristics of the Nile River of Aswan governorate

|  | **Summer** | | | | | | **Winter** | | | | | |
| --- | --- | --- | --- | --- | --- | --- | --- | --- | --- | --- | --- | --- |
|  | **Aswan** | | **Kom Ombo** | | **Edfu** | | **Aswan** | | **Kom Ombo** | | **Edfu** | |
|  | Min. | Max. | Min. | Max. | Min. | Max. | Min. | Max. | Min. | Max. | Min. | Max. |
| **Temp. (^O^C)** | **23.51** | **23.60** | **22.92** | **24.50** | **25.20** | **28.10** | **17.18** | **17.92** | **17.54** | **28.96** | **17.20** | **21.80** |
| **pH** | **7.61** | **7.70** | **7.66** | **7.93** | **7.42** | **7.89** | **7.39** | **7.50** | **7.35** | **8.50** | **7.68** | **7.98** |
| **DO (mg/L)** | **3.82** | **4.01** | **4.10** | **4.53** | **5.10** | **5.50** | **7.55** | **7.67** | **6.03** | **10.31** | **6.10** | **7.50** |
| **Turb (NTU)** | **0.21** | **0.25** | **0.05** | **6.01** | **0.35** | **15.10** | **0.22** | **0.26** | **0.09** | **9.01** | **0.31** | **17.10** |
| **TDS (mg/L)** | **163.5** | **163.9** | **117.1** | **270.1** | **174.3** | **217.7** | **168.1** | **168.5** | **134.6** | **245.4** | **174.2** | **207.8** |
| **TSS (mg/L)** | **1.11** | **1.23** | **0.93** | **4.67** | **3.78** | **13.10** | **0.85** | **1.21** | **1.20** | **6.90** | **2.98** | **9.10** |
| **COD (mg/L)** | **4.97** | **5.10** | **6.69** | **13.10** | **11.30** | **41.30** | **5.90** | **6.01** | **6.02** | **59.6** | **12.70** | **51.10** |
| **BOD (mg/L)** | **2.82** | **3.01** | **3.84** | **7.67** | **7.10** | **27.70** | **2.82** | **3.62** | **3.68** | **36.50** | **8.01** | **35.1** |
| **Phenol (µg/L)** | **ND** | **ND** | **ND** | **ND** | **0.88** | **8.01** | **ND** | **ND** | **ND** | **ND** | **0.02** | **10.10** |
| **TN-N (µg/L)** | **387.5** | **400.7** | **400.2** | **1403.1** | **494.8** | **1127.5** | **402.3** | **420.3** | **480.6** | **3345.4** | **654.1** | **2223.1** |
| **NO_3_-N (µg/L)** | **322.2** | **340.9** | **97.9** | **1000.5** | **389.5** | **570.1** | **342.3** | **360.5** | **112.8** | **1000.4** | **440.1** | **1400** |
| **NH_3_-N (µg/L)** | **36.2** | **45.2** | **26.1** | **74.3** | **20.9** | **30.7** | **45.5** | **50.2** | **20.8** | **150.1** | **44.1** | **120.2** |
| **TP (µg/L)** | **20.5** | **20.9** | **28.9** | **52.4** | **28.5** | **42.4** | **23.2** | **23.7** | **20.5** | **72.7** | **19.6** | **25.1** |
| **Cl (mg/L)** | **6.46** | **6.67** | **7.76** | **27.4** | **8.91** | **17.1** | **6.32** | **6.67** | **7.21** | **8.43** | **9.73** | **17.1** |
| **SO_4_ (mg/L)** | **11.80** | **12.30** | **10.50** | **29.70** | **13.90** | **24.10** | **11.91** | **12.01** | **12.20** | **14.30** | **13.50** | **16.70** |
| **F (µg/L)** | **272.3** | **290.5** | **313.7** | **332.9** | **281.1** | **290.1** | **299.5** | **310.2** | **200.9** | **390.1** | **284.9** | **310.1** |
| **Alkalinity (mg/L)** | **109.1** | **109.5** | **108.4** | **125.3** | **116.2** | **121.3** | **116.5** | **118.2** | **115.6** | **126.1** | **122.5** | **125.8** |
| **Hardness (mg/L)** | **98.6** | **100.4** | **97.6** | **105.7** | **102.2** | **103.7** | **99.9** | **101.7** | **97.6** | **102.3** | **102.8** | **105.5** |
| **WAWQI** | **27.4** | **27.8** | **25.1** | **52.6** | **38.1** | **90.3** | **24.5** | **24.9** | **27.8** | **127.4** | **30.3** | **121.9** |
| **CWQI** | **85.48** | **85.52** | **74.20** | **93.69** | **68.3** | **86.6** | **85.49** | **85.52** | **74.20** | **93.69** | **68.3** | **86.6** |

SM Table 2: Weights used in calculating the WAWQI

| Parameter | Weight |
| --- | --- |
| p H | 0.0637 |
| TDS | 0.0011 |
| DO | 0.0902 |
| COD | 0.0541 |
| BOD | 0.0902 |
| T. N | 0.1546 |
| T. P | 0.2706 |
| SO4 | 0.0027 |
| NO3- N | 0.2706 |
| F | 0.0011 |
| NH3-N | 0.0011 |
